# Ferroptosis in liver disease: new insights into disease mechanisms

**DOI:** 10.1038/s41420-021-00660-4

**Published:** 2021-10-05

**Authors:** Jing Wu, Yi Wang, Rongtao Jiang, Ran Xue, Xuehong Yin, Muchen Wu, Qinghua Meng

**Affiliations:** 1 Department of Medical Oncology, Beijing You-An Hospital, Capital Medical University, Beijing, 100069 China; 2 State Key Laboratory of Proteomics, Beijing Proteome Research Center, National Center for Protein Sciences (Beijing), Beijing Institute of Lifeomics, Beijing, 102206 China; 3 Brainnetome Center and National Laboratory of Pattern Recognition, Institute of Automation, Chinese Academy of Sciences, Beijing, 100190 China; 4 Key Laboratory of Carcinogenesis and Translational Research (Ministry of Education), Department of Gastrointestinal Oncology, Peking University Cancer Hospital & Institute, Beijing, 100036 China

**Keywords:** Liver diseases, Hepatocellular carcinoma

## Abstract

Characterized by excessive iron accumulation and lipid peroxidation, ferroptosis is a novel form of iron-dependent cell death, which is morphologically, genetically, and biochemically distinct from other well-known cell death. In recent years, ferroptosis has been quickly gaining attention in the field of liver diseases, as the liver is predisposed to oxidative injury and generally, excessive iron accumulation is a primary characteristic of most major liver diseases. In the current review, we first delineate three cellular defense mechanisms against ferroptosis (GPx4 in the mitochondria and cytosol, FSP1 on plasma membrane, and DHODH in mitochondria), along with four canonical modulators of ferroptosis (system Xc^−^, nuclear factor erythroid 2-related factor 2, p53, and GTP cyclohydrolase-1). Next, we review recent progress of ferroptosis studies delineating molecular mechanisms underlying the pathophysiology of several common liver diseases including ischemia/reperfusion-related injury (IRI), nonalcoholic fatty liver disease (NAFLD), alcoholic liver disease (ALD), hemochromatosis (HH), drug-induced liver injury (DILI), and hepatocellular carcinoma (HCC). Furthermore, we also highlight both challenges and promises that emerged from recent studies that should be addressed and pursued in future investigations before ferroptosis regulation could be adopted as an effective therapeutic target in clinical practice.

## Facts

1. Iron overload and oxidative stress constitute the major triggers leading to liver injury and disease progression for most liver diseases.
2. As a novel type of iron-dependent cell death, ferroptosis has been gaining much attention in recent years in the settings of liver diseases.
3. Ferroptosis is implicated in the pathophysiology of liver diseases through various signaling pathways.
4. Ferroptosis has a dual role in liver diseases, by aggravating liver damage in chronic liver diseases like IRI, NAFLD, ALD, HH, and DILI, or by increasing the sensitivity to sorafenib in HCC.

## Open questions

1. What are the precise mechanisms that trigger ferroptotic cascade downstream lipid peroxidation?
2. Is there an optimal timepoint when intervening ferroptosis can prevent the progression of chronic liver diseases?
3. Whether the heterogeneity of pathogenesis and treatment, which is common among distinct liver diseases, would affect the process of ferroptosis regulation and the intervention efficiency in clinical practice?
4. Can ferroptotic cell death be ultimately targeted for therapeutic interventions in curing liver diseases?

## Introduction

Ferroptosis was first identified in 2012 as a novel form of regulated cell death (RCD). It is characterized by excessive accumulation of intracellular lipid reactive oxygen species (ROS) and lipid peroxidation resulting from iron-dependent depletion of glutathione (GSH) and the inactivation of glutathione peroxidase 4 (GPx4). This new mode of cell death is morphologically, genetically, and biochemically distinct from other well-known cell death including apoptosis, necroptosis, pyroptosis, and autophagy [1].

Tremendous effort has been devoted to examining the physiological and pathophysiological effects of ferroptosis, which has dramatically expanded our knowledge. Indeed, ferroptotic cell death was reported before the inception of ferroptosis but was ascribed to apoptosis and other types of cell death [2]. Core events of ferroptosis involve increased iron accumulation, impaired lipid repair systems, and lipid peroxidation, which culminate in membrane destruction and cell death [3]. Cells undergoing ferroptosis generally exhibit abnormal mitochondrial morphology, including mitochondrial shrinkage, membrane density increase, and rupture of the outer mitochondrial membrane [4]. Despite lacking established criteria signifying the occurrence of ferroptosis, the three most commonly reported signatures are lipid peroxidation, increased prostaglandin synthase 2 (PTGS2) expression, and decreased content of the reduced form of nicotinamide adenine dinucleotide phosphate (NADPH) [3, 5, 6].

Mounting evidence has indicated that ferroptosis is involved in several pathophysiological contexts including cancer [7], neurodegeneration [8], heart disease [9], and in particular, liver diseases [10]. Increasing attention has been paid to the role of ferroptosis in liver diseases partially because iron overload has been observed in many contexts of these diseases.

In this review, we will focus on ferroptosis studies in the settings of liver diseases. We first outline three cellular defense mechanisms against ferroptosis, alongside four internal modulators of ferroptosis, and how each of them participates in ferroptosis. Next, we highlight the involvement and underlying mechanisms of ferroptosis in several specific liver diseases, including ischemia/reperfusion-related injury (IRI), nonalcoholic fatty liver disease (NAFLD), alcoholic liver disease (ALD), hemochromatosis (HH), drug-induced liver injury (DILI), and hepatocellular carcinoma (HCC). Finally, challenges and future directions regarding the investigation of ferroptosis are discussed.

## Three distinctive cellular protective mechanisms against ferroptosis

Studies have hitherto demonstrated that cells may have evolved at least three defense systems with different subcellular localizations, of which cytosol- and mitochondria-localized GPx4 confers the major protection against lipid peroxidation, while ferroptosis suppressor protein 1 (FSP1) acts mainly on the plasma membrane, and dihydroorotate dehydrogenase (DHODH) is a major defensive arm in mitochondria.

## GPx4-mediated ferroptosis resistance

GPx4, a GSH-dependent selenium-containing enzyme, is the only enzyme in lipid hydroperoxides scavenger that can convert toxic lipid hydroperoxides to their corresponding nontoxic lipid alcohols. It performs an essential biological function given existing evidence demonstrating that GPx4-knockout is embryonic lethality at an early stage [11]. In addition, deletion of GPx4 could result in massive cell death and cell degeneration (e.g., hepatocyte and neurons), which could be rescued by lipophilic antioxidant vitamin E and ferroptosis inhibitors [11]. A further step in understanding the function of GPx4 has recognized that there is a generality of ferroptosis regulation by GPX4 (in the cytosol and mitochondria), which constitutes the cornerstone of the defensive system in response to lipid peroxidation. Genetic or pharmacologic inactivation of GPx4 can induce a characteristic ferroptosis death [3]. Specifically, GPx4-regulated ferroptosis participates in cancer initiation and progression. In addition, drug-tolerant persister cells are highly dependent on GPx4 for survival, and loss of GPx4 function contributes to ferroptotic cell death and prevents drug resistance, suggesting a novel therapeutic avenue in cancers [12]. For neurodegenerative diseases, GPx4 ablation-induced ferroptosis accounts for cognitive impairment and neurodegeneration, which could be ameliorated by ferroptosis inhibitors [13]. In the context of acute or chronic tissue/cell injury, inhibiting ferroptosis via adaptively upregulating GPx4 is frequently regarded as a protective mechanism against various unfavorable factors [14]. Overall, GPx4 plays a critical role in the regulation of ferroptosis, affecting the development and progression of multiple diseases.

## FSP1-related system

GPx4 is widely accepted as a primary regulator of ferroptosis [15], however, the sensitivity of cells to GPx4 inactivation-induced ferroptosis varies greatly across cancer types [16]. Thus, alternative mechanisms may exist in parallel to GPx4 to confer ferroptosis resistance. FSP1 is a newly identified endogenous potent ferroptosis suppressor, which confers ferroptosis resistance through reducing coenzyme Q_10_. Specifically, the myristoylation of FSP1 enables its location to the plasma membrane and further suppresses phospholipid peroxidation by regenerating a reduced form of coenzyme Q_10_ (CoQ_10_-H2) using NAD(P)H [17, 18]. The expression of FSP1 is positively associated with the resistance of cells to GPx4 inhibitors, and FSP1 is essential to maintain tumor cell growth in the absence of GPx4 [17]. These investigations outlined a distinct mechanism to cope with ferroptosis and implied that FSP1 may serve as a therapeutic target for personalized cancer therapy.

## DHODH-mediated ferroptosis defense

DHODH is an inner mitochondrial membrane enzyme, which can catalyze the synthesis of the de novo pyrimidine ribonucleotide. Due to an increased demand for nucleotides in rapidly proliferating cells, DHODH has been intensively explored as a promising target for cancer therapy during the past years [19, 20]. A recent study using global metabolomic analysis in conjunction with metabolic tracer analysis suggested a distinctive function of DHODH in mitigating mitochondrial lipid peroxidation and thus ferroptosis, which was independent of its conventional role in producing pyrimidine nucleotides [21]. Furthermore, this study discovered that the inactivation of DHODH could sensitize GPX4^high^ cancer cells to ferroptosis inducers, and potentiate ferroptosis in GPX4^low^ cancer cells. Remarkably, DHODH acts in parallel to GPX4 in regulating ferroptosis in mitochondria, but independently of cytosolic GPX4 or FSP1. Mechanically, this novel action of DHODH may result from reducing ubiquinone to ubiquinol [22]. Promisingly, this study indicated that DHODH inhibitors treatment either alone in GPX4^low^ cancers, or in combination with ferroptosis inducers in GPX4^high^ cancer could confer tumor inhibition [21]. These results add to the improved understanding of the DHODH in ferroptosis regulation.

## Several canonical ferroptosis modulators

### System Xc^−^

**Fig. 1:**
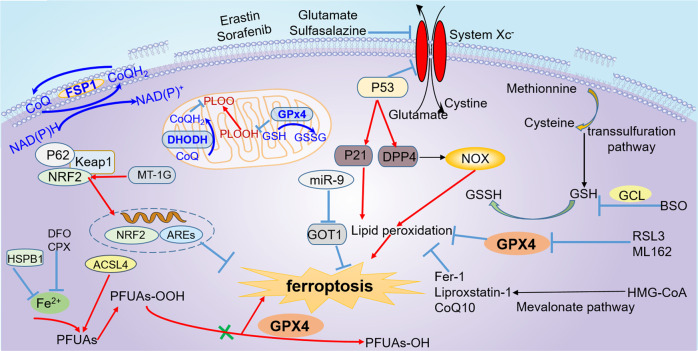
Summary of key regulators and related mechanisms of ferroptosis. The three primary defense systems include GPx4-mediated ferroptosis resistance, FSP1-related system, and DHODH-mediated ferroptosis defense. The regulators that participate in the regulation of ferroptosis mainly involve Nrf2, SLC7A11, P53, and GCH1. Other regulators and potential mechanisms related to ferroptosis are also shown here. GPx4 glutathione peroxidase 4, FSP1 ferroptosis suppressor protein 1, DHODH dihydroorotate dehydrogenase, Nrf2nuclear factor erythroid 2-related factor 2, GCH1 GTP cyclohydrolase-1.

System Xc^−^ is a chloride-dependent membrane cystine/glutamate antiporter that generally imports cystine and exports glutamate with 1:1 stoichiometry. It is a heterodimeric protein containing a light chain SLC7A11 (xCT, a 12-pass transmembrane transporter protein), and a heavy chain SLC3A2 (4F2 heavy chain, a transmembrane regulatory subunit). Inhibiting the antiporter can result in the depletion of intracellular cysteine, hampering GSH synthesis, which further impedes the function of GPx4 and triggers ferroptosis (Fig. 1).

In addition to pharmacological inhibition, SLC7A11 is a genetic target of various endogenous proteins in inducing ferroptosis [23]. For example, the tumor suppressor p53 could sensitize cells to ferroptosis by repressing the expression of SLC7A11 (Fig. 1) [24]. SLC7A11 is upregulated in several human tumor tissues and may function as an oncogene. Moreover, overexpression of SLC7A11 can suppress ferroptosis and counteract p53-related tumor suppression [24]. Notably, deleting SLC7A11 in noncancerous cells was insufficient to induce ferroptosis but can exacerbate the cell’s susceptibility to iron overload-induced ferroptosis in a mice model of HH [25]. This study suggested that SLC7A11 may be a clinically viable target for both therapeutic potential and safety.

### Nrf2

The nuclear factor erythroid 2-related factor 2 (Nrf2) is an important transcription factor that can protect cells against various oxidative and toxic insults by sensing and integrating diverse environmental cues to regulate cytoprotective genes. Nrf2 participates in diverse biological processes, including antioxidant response, iron metabolism, proteostasis, keeping normal mitochondrial function, and apoptosis [26]. In recent years, the role of Nrf2 in regulating ferroptosis has been increasingly recognized, and evidence is rapidly accumulating. Under basal conditions, the expression of Nrf2 remains low due to the proteasomal degradation mediated by the Cul3/Rbx1 E3 ubiquitin ligase. In response to oxidative stress, Nrf2 is activated upon release from the Kelch-like ECH-associated protein 1 (Keap1). Activated Nrf2 translocates to the nuclear and subsequently upregulates the expression of a plethora of genes bearing antioxidant response elements (AREs), many of which are responsible for protecting against lipid peroxidation. Besides, Nrf2 plays essential roles in metabolism regulation (e.g., glucose metabolism), and the metabolites produced in these processes including NADPH and GSH, help maintain redox balance. Furthermore, Nrf2 is an upstream regulator of many vital genes of the ferroptotic cascade including GPx4 and System Xc^−^ [27]. Therefore, Nrf2 is considered a negative regulator of ferroptosis and promoter of ferroptosis resistance.

For tumor cells, pathways involving Nrf2 activation are frequently responsible for decreased sensitivity to ferroptosis, which confer resistance to anticancer drugs [28, 29]. For example, in HCC, activation of the p62-Keap1-Nrf2 pathway prevents Nrf2 degradation and consequently protects HCC cells against ferroptosis. Genetic or pharmacological suppression of Nrf2 expression could increase sorafenib’s anticancer activity in both vitro and tumor xenograft models [7]. While in other stress-induced diseases, ferroptosis inhibition resulting from Nrf2 activation helps protect against oxidative stress and maintain tissue homeostasis [27, 30, 31]. Consequently, targeting Nrf2-regulated ferroptosis as a therapeutic strategy should take specific pathological contexts into account.

### p53

p53 is a classical transcription factor that can prevent cancer development by regulating the cell cycle, senescence, apoptosis, and energy metabolism. Consequently, it is widely known as a tumor suppressor. A growing body of literature underscores that ferroptosis is one of the mechanisms through which p53 exerts antitumor activities. This non-canonical tumor suppressor function is independent of cell growth arrest, apoptosis, and senescence. Specifically, Gu et al. reported that p53 increased cell sensitivity to ferroptosis through repressing the expression of SLC7A11 and thus cystine uptake, which could be reversed by ferroptosis inhibitor fer-1 and SLC7A11 overexpression [24]. Notably, p53^3KR^ (K117R + K161R + K162R), acetylation–defective mutant, preserved this effect of sensitizing cells to ferroptosis. Importantly, later results from this group found that p53^4KR^ (K98R + 3KR) was unable to repress SLC7A11 and displayed a phenotype of ferroptosis resistance [32]. This body of work highlights that acetylation is indispensable for p53-mediated ferroptosis and tumor suppression [32].

However, such findings were not convergent across all extant investigations. In some conditions, p53 was discovered to negatively regulate ferroptosis. For example, Xie et al. observed that p53 limited erastin-induced ferroptosis by promoting the nuclear accumulation of dipeptidyl peptidase-4 (DPP4) and further increasing the expression of SLC7A11 in human colorectal cancer [33]. Coincidentally, Dixon et al. pointed out that the p53-p21 axis could delay the onset of ferroptosis induced by cysteine deprivation in human Ht-1080 fibrosarcoma cells, which facilitated the adaptation of cancer cells to metabolic stress [34]. Collectively, this bidirectional regulation of ferroptosis by p53 is complex and largely acts in a cell-type dependent and context-specific manner. These findings open exciting cues to systematically explore the unique role of p53-dependent tumor suppression based on ferroptosis.

## GTP cyclohydrolase-1 (GCH1)

GCH1, the rate-limiting enzyme for biosynthesis of tetrahydrobiopterin (BH4), is recently identified as another novel molecule counteracting ferroptosis using a whole-genome activation screen. GCH1 is overexpressed in response to all of the three ferroptosis inducers (RSL3, erastin, and genetic ablation of Gpx4). Increased GCH1 promotes the biosynthesis of BH4/BH2 folate and reduced CoQ_10_, and these metabolites are potent antioxidants to hamper the process of lipid peroxidation. Notably, GCH1 selectively protects phospholipids with two polyunsaturated fatty acyl tails from degradation [35]. Similar to FSP1, there is a clear correlation between GCH1 expression and the resistance of cancer cells to ferroptosis, indicating that the GCH1-BH4-phospholipid axis may be a potential target in tumor therapy.

## Some other regulators

Apart from the four regulators mentioned above, some other regulators have attracted researchers’ attention. For example, miR-9 could reduce erastin- and RSL3-induced ferroptosis via repressing glutamate oxaloacetate transaminase 1 (GOT1) in melanoma, and this effect could be rescued by inhibiting the glutaminolysis process [36]. Heme oxygenase-1 (HO-1), a cytoprotective enzyme, was upregulated upon treatment with erastin or RSL3 in renal proximal tubule cells, while deletion of HO-1 increased the cellular susceptibility to erastin-induced ferroptosis [37]. Other potential regulators and pathways of ferroptosis, such as miR-137 [38], peroxiredoxin-6 (PRDX6) [39], activating transcription factor 4 (ATF4) [40], heat shock protein beta 1(HSPB1) [41], the mevalonate pathway, and the transsulfuration pathway [42] are emergent players in ferroptosis regulation (Fig. 1).

## Pharmacologic regulation of ferroptosis

Compounds precisely modulating ferroptosis (inducers or inhibitors) play vital roles in elucidating the mechanisms of ferroptosis-related diseases. To date, the best-studied and most broadly-used inducers can be categorized into two classes. Class I ferroptosis stimulants are compounds targeting system Xc^−^ (e.g., erastin [43], sorafenib [7], sulfasalazine [44], and glutamate [43, 45]), while inhibitors directly targeting GPx4 (e.g., RAS selective lethality protein 3 [RSL3], ML-162) are considered as Class II ferroptosis inducers [3, 46, 47]. In some cases, some studies also applied other compounds to trigger ferroptosis [21, 48–51] (Table 1). Concerning inhibitors, fer-1 [43, 52], liproxstatin-1 [52, 53], and deferoxamine (DFO) [43] are the three most frequently used compounds, while other less frequently used drugs also exhibit the property of ferroptosis repression [17, 33, 54–59] (see Table 2).

**Table 1:** Major inducers of ferroptosis.

	Reagent	Function	Reference
Class I inducers	Erastin	Target system Xc^−^ and deplete GSH	[43]
	Sorafenib	Inhibit cystine uptake through system Xc^−^ and deplete GSH	[7]
	Sulfasalazine	Inhibit cystine uptake through system Xc^−^ and deplete GSH	[44]
	Glutamate	High concentrations of extracellular glutamate inhibit cystine uptake through system Xc^−^	[43, 45]
Class II inducers	RSL3	Inhibit GPx4 and cause argument of lipid hydroperoxides	[3, 47]
	ML-162	Inhibit GPx4 and cause argument of lipid hydroperoxides	[46]
Other types of inducers	FIN 56	Decrease GPx4 abundance; bind to and activate squalene synthase and suppress CoQ_10_	[48]
	Dihydroartemisinin (DHA)	Might decrease the expression of GPx4	[49]
	Ferric ammonium citrate (FAC)	Cause iron overload and lipid peroxidation	[43]
	l-buthionine (S, R)-sulfoximine (BSO)	Inhibit glutamate-cysteine ligase (GCL) and deplete GSH	[51]
	Trigonelline	Inhibit Nrf2	[7]
	FINO(2)	Indirectly inhibit GPx4 enzymatic function and directly oxidize iron, induce broad lipid peroxidation	[50]
	Brequinar	Inhibit DHODH and thus decrease the reduction of ubiquinone to ubiquinol	[21]

**Table 2:** Major inhibitors that modulate ferroptosis.

Reagent	Function	Reference
Fer-1	Prevent accumulation of cytosolic and lipid ROS as well as inhibit lipid peroxidation, serving as a lipid ROS scavenger	[43, 52]
Liproxstatin-1	Function as radical-trapping antioxidants (RTAs) to inhibit lipid peroxidation	[52, 53]
Lipophilic antioxidant (vitamin E, Trolox)	Damp lipoxygenase (LOX), and abrogate hydroxyl group radicals	[56]
DFO, ciclopirox olamine (CPX)	Reduce intracellular iron levels and inhibit lipid peroxidation	[43]
Selenium, sodium selenite	Increase the levels of GPx4	[54]
CoQ_10_	Act as a lipophilic radical-trapping antioxidant to halt the propagation of lipid peroxides	[17]
β-mercaptoethanol (β-ME)	Reduce cystine to cysteine thus circumvent the inhibition of system Xc^−^	[43]
Ethyl 3-(benzylamino)-4-(cyclohexylamino) benzoate (SRS11-92)	Block accumulation of lipid hydroperoxides possibly through inhibiting LOX and its radical-trapping properties	[52, 57]
Baicalein	Target lipoxygenases and repress lipoxygenase-triggered lipid peroxidation	[58]
XJB-5-131	Have an antioxidant effect and suppress lipid peroxidation	[59]
Vildagliptin, alogliptin, linagliptin	Inhibit DPP4 and block DPP4-induced lipid peroxidation	[33]

## The involvement of ferroptosis in typical liver diseases

Iron overload and oxidative stress constitute the top two most important triggers leading to liver injury and disease progression in most liver diseases. Characterized by iron overload and lipid peroxidation, ferroptosis has been broadly reported to be involved in multiple liver diseases. The rest of this review will focus on summarizing the role of ferroptosis in the pathophysiology of several common liver diseases (see Supplementary Table 3).

### IRI

IRI is a pathological state that occurs in several organs exerting local and systemic adverse effects. Hepatic IRI is a major problem for liver transplantation. Accumulating evidence indicates that ferroptosis is involved in the pathogenesis of IRI, and targeting ferroptosis may be a promising therapeutic approach [60]. GPx4 inactivation-induced ferroptosis is supposed to be a contributor to IRI-induced liver injury, and ferroptosis inhibitor liproxstatin-1 could reverse liver injury and improve liver function [53]. Iron overload, lipid peroxidation, and upregulation of the ferroptosis indicator PTGS2 are typical characteristics of the liver undergoing hepatic IRI. Notably, fer-1 could improve the inflammatory response and liver injury [53, 61].

### NAFLD

The incidence of NAFLD is rapidly increasing, which has become a leading cause of chronic liver disease. Both nature and nurture risk factors could make an individual being more prone to develop NAFLD [62]. Although tremendous effort has been devoted to NAFLD, the underlying mechanism remains elusive. It is widely accepted that iron overload is common in patients with NAFLD, and iron-induced lipid peroxide is one of the major contributors to NAFLD [63, 64]. In addition, an iron imbalance is implicated in obesity and insulin resistance [65], both of which are typical characteristics of NAFLD patients. Furthermore, Nrf2 regulates fatty acid synthesis via suppressing enzyme expression and slows down NASH development through the p62-Keap1-Nrf2 axis [66]. Collectively, it is tempting to speculate that ferroptosis may be implicated in the pathogenesis of NAFLD, which has been substantiated by a plethora of studies.

As a concrete example, ferroptosis, instead of necroptosis, was verified as the major type of RCD to initiate cell death and inflammation in steatohepatitis [67]. Loguercio et al. observed that more than 90% of NAFLD patients enrolled in their study exhibited elevated levels of lipid peroxidation markers (malondialdehyde [MDA] and 4-hydroxynonenal [4-HNE]). In particular, the MDA and 4-HNE were much higher in NASH patients than those in steatosis patients [68]. Remarkably, Qi et al. observed that RSL3 treatment can aggravate manifestations (levels of serum biochemical parameters, hepatic steatosis, and inflammation) of methionine/choline-deficient (MCD) diet-induced NASH mice, while administration of sodium selenite (a GPx4 activator), deferoxamine mesylate salt, or liproxstatin could rescue RSL3 induced lipid peroxidation and cell death as well as the NASH severity [54]. Similarly, Li et al. demonstrated that arachidonic acid metabolism could trigger ferroptosis in the MCD diet-induced NASH mice model, suggesting that targeting ferroptosis could attenuate MCD diet-induced steatosis, inflammation, and fibrosis [69]. Consistently, other investigations also discovered that some drugs like Ginkgolide B and dehydroabietic acid have beneficial effects on alleviating NASH severity through inhibiting ferroptosis. In these contexts, Nrf2 and GPx4 stand out as the major protective mechanisms [70, 71]. Overall, these results implied that the regulation of ferroptosis in the context of NAFLD is an intriguing notion that deserves further investigation.

### ALD

Although there is a growing consensus that lipid disorder and hepatic iron overload are prevalent features of ALD [72], only in recent years have researchers began taking ferroptosis’ role in alcoholic liver damage into consideration. During the repair of ethanol-induced liver damage, ferroptosis is always negatively regulated [73, 74]. Alcohol treatment results in massive ROS accumulation and lipid peroxidation both in cell culture and in mouse models of ALD, which could be rescued by a ferroptosis inhibitor [74]. Similarly, some investigations found that dimethyl fumarate has a protective effect on ethanol-induced oxidative injury through activating the Nrf2 pathway and repressing ferroptosis [73]. Moreover, a recent study implied that ferroptosis was the downstream event of some genes like frataxin to induce liver injury in the context of excessive alcohol exposure [75].

Ferroptosis was also implicated in the ALD development through the well-characterized crosstalk between the liver and other organs (e.g., gut, adipose, brain, and lung), among which the intestine–liver and liver–adipose interactions are most frequently reported [76]. Specifically, Zhou et al. discovered that the deficiency of intestinal sirtuin 1 could normalize alcohol-induced iron imbalance and limit the onset of ferroptosis [77], ultimately leading to the amelioration of ethanol-induced liver injury. Another investigation also confirmed the linkage between ferroptosis regulation and adipose-liver axis in alcoholic steatohepatitis [78]. Specifically, this study observed that adipose-specific lipin-1 overexpression exacerbated alcoholic steatohepatitis and hepatobiliary damage via aggravating abnormal iron deposition and increasing hepatic MDA levels in a GPx4-independent manner [78]. Looking ahead, extensive investigations aiming at exploring notable therapeutic targets based on ferroptosis for patients with ALD are encouraged in the future.

### HH

Hemochromatosis is an inherited disease caused by genetic mutations impairing iron metabolism [79]. As a result, excessive iron is absorbed by the intestine and deposited in parenchymal cells, leading to tissue damage and organ failure [79]. Apart from genetic factors, environmental aspects like alcohol intake and blood loss can also influence iron accumulation. Excessive iron usually produces massive ROS through the Fenton reaction and subsequently leads to DNA damage and tissue injury.

A wealth of research has reached the consensus that iron overload has a close relationship with liver injury and disease progression of HH [80]. However, whether and how ferroptosis participates in the pathogenesis of HH is still unknown. It has only been within the last few years that researchers have started to conduct some preliminary investigations to examine this association. For example, Wang et al. demonstrated that ferric citrate triggered ferroptosis both in cell culture and in mice with the presentence of ferroptosis signatures, evidencing the involvement of ferroptosis in HH [25]. More importantly, this study further showed that SLC7A11 as a candidate gene of ferroptosis in HH, and suggested that the ROS-Nrf2-ARE axis was responsible for the upregulated SLC7A11, which may be a possible compensatory mechanism to protect against iron overload-induced ferroptosis in HH [25].

Given that iron overload is the most typical characteristic of HH, there should be mounting evidence available illuminating the association between ferroptosis and HH. Unfortunately, such studies are really limited. A possible reason is that most of these studies focusing on examining the association between HH and iron overload were conducted before “ferroptosis” was first proposed. Therefore, it deserves further investigation to delineate the specific function and mechanism of ferroptosis in HH.

### DILI

The DILI can cause a spectrum of liver damages ranging from aberrant transaminases to life-threatening acute liver failure [81]. There are two subtypes of hepatotoxicity, one being the direct, dose-dependent toxicity (also known as intrinsic type) and the other being idiosyncratic toxicity (representing an unpredictable injury with a genetic predisposition). Idiosyncratic hepatotoxicity is less common and only affects specific populations. Acetaminophen (APAP)-induced intrinsic DILI is more widely reported in clinical settings. Thus far, APAP is the most common drug used to establish DILI mice models, given evidence demonstrating a shared mechanism of APAP toxicity between humans and mice [82].

GSH is essential in the detoxification of *N*-acetyl-p-benzoquinone imine (NAPQI, a highly reactive metabolite of APAP). Consequently, a decrease in GSH and inhibition of GPx4 are common in APAP-induced cell death [83]. These findings support the hypothesis that ferroptosis may be a critical participant in DILI, which is corroborated by recent investigations. For example, Lőrincz et al. found that fer-1 increased the viability of primary mouse hepatocytes in the presence of APAP in vitro [84]. However, fer-1 did not restore the cellular GSH level, implying that ferroptosis may participate in the APAP-induced cell death, but the protective effect of fer-1 could not be ascribed to the suppressed conversion from APAP to NAPQI. Similarly, a recent study observed that APAP-treated mice would exhibit increased ferroptosis symptoms [85]. Consistently, when treated with fer-1, the above events were significantly reduced, alongside a decreased alanine aminotransferase/aspartate aminotransferase ratio (ALT/AST) ratio, improved histopathological conditions, and reduced mortality of mice. In addition, abrogation of acyl-CoA synthetase long-chain family member 4 (ACSL4), a key lipid metabolism enzyme that can promote ferroptosis, could also relieve hepatotoxicity and lipid peroxidation [85]. Similarly, some other studies observed the elevation of MDA and the reduction of GSH in APAP-treated hepatocytes [86]. Some researchers noted that dietary contents high in polyunsaturated fatty acids (PUFA) might increase hepatocytes’ susceptibility to lipid peroxidation [87]. However, Yamada et al. suggested that auto-oxidation is the predominant form to promote lipid oxidation in APAP-induced hepatotoxicity [85]. All these results indicated that ferroptosis might get involved in APAP-induced hepatocyte cell death.

Despite the above findings, whether ferroptosis is involved in the pathology of DILI is a highly contentious and controversial subject of debate. For example, Knight et al. reported that α-tocopherol could not improve APAP-induced liver injury and lipid peroxidation was not a contributor to APAP-triggered hepatotoxicity [88]. Furthermore, a recent review argued that APAP-induced hepatotoxicity should be identified as programmed necrosis, while other types of cell death could not fit the characteristic of the toxicity of APAP [89]. Based on this evidence, many efforts are required before ferroptosis is formally recommended as a therapeutic target for DILI.

### HCC

There is a growing consensus that ferroptosis provides a novel tumor suppression mechanism [46, 90, 91]. In terms of HCC, existing studies primarily focused on sorafenib-induced ferroptosis and its potential targets. Sorafenib is a multikinase inhibitor that is widely used in the treatment of advanced HCC [23]. Multiple critical genes are involved in the sorafenib-induced ferroptosis and subsequent tumor repression. For example, the retinoblastoma (Rb) protein is responsible for cell proliferation and cell cycle regulation through interacting with members of the E2F family of transcription factors. The Rb loss-of-function is a common event in tumorigenesis. Louandre et al. demonstrated that inactivation of Rb could increase sorafenib’s efficacy both in vitro and in murine xenografts of HCC through aggravating ferroptosis [92]. Mechanistically, this study suggested that Rb did not affect GSH metabolism and GPx4 expression in the presence of sorafenib; instead, the therapeutic effect arose from the massive ROS produced by the mitochondria.

Although sorafenib remains the first-line systemic therapy for advanced HCC patients, drug resistance is a significant issue that limits its broad application. Genes negatively regulating ferroptosis could confer cells the ability of drug-resistance. For example, Sun et al. found that sorafenib treatment could induce the expression of Metallothionein-1G (MT-1G) in an Nrf2-dependent manner, which further contributed to the sorafenib resistance [93]. Upregulation of MT-1G suppressed sorafenib-induced ferroptosis, indicating that MT-1G may function as a negative regulator of ferroptosis in HCC [93]. As mentioned above, erastin or sorafenib treatment can activate Nrf2 via the P62-keap1 pathway, enabling the activation of a series of target genes capable of eliciting detoxification and antioxidation response, which contributes to ferroptosis resistance [7]. The knock down of Nrf2 significantly inhibited the growth of HCC cells. CDGSH iron sulfur domain 1 (CISD1), another negative regulator of ferroptosis in HCC, was found to be upregulated upon erastin treatment. Genetic inhibition of CISD1 promoted erastin-induced ferroptotic cell death through repressing mitochondrial lipid peroxidation in a GPx4-independent manner [94]. This finding concurs with the observation that mitochondria is critical in cysteine deprivation-induced ferroptosis but not in GPx4 repression-induced ferroptosis [95]. Fortunately, some studies found that haloperidol (a sigma receptor 1 antagonist) can facilitate the cascade of ferroptotic cell death induced by sorafenib in HCC. Potentially most intriguing is that this evidence provides a novel possibility that haloperidol may have the potency to be used in combination therapy for HCC by reducing sorafenib dosage and overcoming drug resistance [96].

Through multiple related molecules and pathways, lipid metabolism could also regulate the ferroptotic response in HCC. Natural omega-3 PUFAs are the predominant peroxide substrates in ferroptosis and possess well-characterized mechanisms of antitumorigenesis [97]. However, whether they still maintain the benefits when the tumor has been established remains largely unknown. A preliminary study engineered a low-density lipoprotein nanoparticle reconstituted with the natural omega-3 PUFA, docosahexaenoic acid (LDL-DHA), and found that this nanoparticle promoted tumor suppression through triggering lipid peroxidation and ferroptosis via suppressing GPx4 activity and disturbing the balance of redox couples in HCC [98]. ACSL4 has been identified as an essential factor in predicting the sensitivity of cells to ferroptosis. Specifically, the expression of ACSL4 is elevated in ferroptosis-sensitive cells compared with that in ferroptosis-resistance cells, and patients who have complete or partial responses to sorafenib have higher liver ACSL4 expression than those who have a poor response to sorafenib [99, 100]. Notably, growing evidence pinpointed that ACSL4 can serve as a potential prognostic factor for overall survival and disease-free survival time [101, 102], suggesting that ACSL4 might be a therapeutic target for HCC patients, especially for those at an advanced stage posttreatment with sorafenib.

Apart from a therapeutic potential, there has been evidence demonstrating the utility of ferroptosis for the prognostic prediction of HCC. For example, a recent investigation identified a novel ferroptosis-related gene signature by leveraging a machine learning-based model to stratify patients into two risk groups [103]. More importantly, this signature can be used for prognostic prediction in HCC, evidenced by the fact that patients in the high-risk group showed significantly reduced overall survival compared with patients in the low-risk group.

## Challenges and future directions

The last few years have registered a significant improvement in our understanding of the role of ferroptosis in the development and pathogenesis of liver diseases. Despite such advances, there remain some challenges that should be overcome before ferroptosis regulation could be adopted as a useful therapeutic tool in clinical practice. Here, we present some challenges as follows.

(I) The available evidence is not sufficiently robust to draw definitive conclusions regarding whether intervening in ferroptosis is overall beneficial at the cellular level. The primary reason is that the amount of research currently being performed on ferroptosis is limited, given that ferroptosis was only first identified in 2012. Although some molecules or factors have long been investigated and reported to participate in ferroptosis regulation, the recognition of their specific role in ferroptosis is fairly recent [12, 23]. Moreover, further investigations are warranted to find a suitable molecule that could regulate ferroptosis and further exert beneficial effects on patients with liver diseases such as HCC.

(II) The vast majority of existing investigations were conducted in animal models or in vitro, while clinical trials involving ferroptosis regulation are scarce [93, 96]. Compelling evidence generally involves the interaction and integration of information from experimental models and patient findings. Therefore, to draw firm conclusions, further studies in humans with randomized trial designs are warranted.

(III) Early efforts characterizing ferroptosis have predominately focused on examining characteristic hallmarks, while less is known about the exact mechanisms that drive the phenotype [83]. This is of great importance because only a systematic approach that integrates functional and mechanistic studies can prove fruitful in identifying the precise role of ferroptosis in certain liver diseases. Furthermore, well-planned translational investigations are in urgent need to provide valuable guidance for clinical applications.

(IV) Although many researchers have devoted themselves to uncovering the precise mechanisms of ferroptosis and have made some achievements, the existing studies mostly focused on the upstream pathways of lipid peroxidation, leaving largely unexplored the downstream molecular pathways or key molecular junctions after the initiation of ferroptosis.

(V) There has been a growing appreciation for the involvement of RCD in liver diseases. In many cases, these cell death types coexist in certain pathological conditions like NAFLD [54, 104, 105], functioning as the backup mechanism to compensate when a major RCD is prevented. A case in point is that Bax/Bak double-knockout (DKO) mice were viable, while Atg5/Bax/Bak triple-knockout (TKO) mice developed embryonic lethality, suggesting that autophagy can compensate for the apoptosis deficiency in embryonic development [106]. Similarly, studies have highlighted the interplay between autophagy and ferroptosis, where autophagy could promote ferroptosis through excessive iron accumulation and system Xc^−^ inhibition, and deleting Atg5 and Atg7 limited erastin-induced ferroptosis [107, 108]. The complex crosstalk between these RCDs complicated the efforts of clearly depicting the role of any specific type of cell death in liver diseases.

(VI) In chronic liver disease (e.g., ALD, NAFLD), ferroptosis is primarily responsible for liver damage, and inhibiting ferroptosis would reverse the deleterious effect. However, in HCC, ferroptosis could increase cell sensitivity to sorafenib, and inhibiting this cell death contributes to drug resistance [54, 74, 85]. Generally, chronic liver injury and repair would end up with fibrosis and even HCC. We wonder if there is an optimal timepoint when intervening in ferroptosis can prevent the progression of chronic liver disease to HCC. In addition, distinct liver diseases have great heterogeneity regarding pathogenesis and treatment. Therefore whether the process of ferroptosis regulation, even its intervention efficiency in clinical practice, is impervious to this heterogeneity should also be considered in future studies.

Given all these various aspects together, there is still a paucity of information to explicitly illuminate how ferroptosis participates in liver diseases so much that this field is fraught with uncertainties. Answers to these questions will further illuminate the ferroptosis pathway and open attractive cues to systematically explore the benefit of ferroptosis therapeutic interventions in curing liver diseases.

## Conclusions

As a new form of cell death, ferroptosis is involved in the pathophysiology of multiple liver diseases. Identifying regulators of ferroptosis would benefit investigators to better understand the mechanisms of ferroptosis-related liver diseases. In the current study, we summarized three cellular defense mechanisms against ferroptosis and four canonical regulators of ferroptosis. We highlighted the involvement of ferroptosis in several typical liver diseases and discussed the potential mechanisms. Specifically, we found that ferroptosis contributes to liver damage in IRI, NAFLD, ALD, HH, and DILI, while in HCC, ferroptosis can increase cell sensitivity to sorafenib. The increased understanding of ferroptosis enables us to interfere with key regulators to inhibit or induce this process, ultimately dampening liver disease progression and paving the way for new drugs.

## Supplementary Information

Supplementary table 3
