# Supplementary material for: Ferroptosis in liver disease: new insights into disease mechanisms
Source: Cell Death Discov. 2021 Oct 5;7:276. doi: 10.1038/s41420-021-00660-4 (PMC8492622; doi:10.1038/s41420-021-00660-4)
Supplement: Supplementary file 1 — Supplementary table 3 [file 41420_2021_660_MOESM1_ESM.docx]

| Table 3. The role of ferroptosis in liver diseases. | | |
| --- | --- | --- |
| **Disease** | **Status of ferroptosis** | **Related references** |
| IRI | Ferroptosis is supposed to be a contributor to IRI-induced liver injury, and ferroptosis inhibition could reverse liver injury and improve liver function. | ^[53,61]^ |
| NAFLD | Signatures of lipid peroxidation are observed in NAFLD patients, especially in NASH patients. Induction of ferroptosis is responsible for aggravated liver damage. | ^[54,68]^ |
| ALD | Repressing ferroptosis exerts a protective effect on ethanol-induced oxidative injury. In addition, ferroptosis is also a target in the liver-organ crosstalk in the development of ALD. | ^[73,77,78]^ |
| HH | Ferroptotic cascade is induced in the context of HH, and ROS-Nrf2-ARE axis may serve as a compensatory mechanism to protect against the iron overload-induced ferroptosis. | ^[25]^ |
| DILI | Ample evidence indicated that ferroptosis inhibition could relieve APAP-induced hepatotoxicity and lipid peroxidation, although whether ferroptosis is involved in the pathology of DILI is still a controversial issue. | ^[85,86,89]^ |
| HCC | Ferroptosis inhibition induced by multiple related molecules and pathways confers sorafenib resistance and tumor progression. In addition, ACSL4-related to lipid metabolism has been identified as an essential factor to predict sensitivity to ferroptosis in HCC. | ^[93,99,100]^ |

**Reference**

25. Wang H, An P, Xie E, Wu Q, Fang X, Gao H, et al. Characterization of ferroptosis in murine models of hemochromatosis. Hepatology. 2017;66:449–65.

53. Friedmann Angeli JP, Schneider M, Proneth B, Tyurina YY, Tyurin VA, Hammond VJ, et al. Inactivation of the ferroptosis regulator Gpx4 triggers acute renal failure in mice. Nat Cell Biol. 2014;16:1180–91.

54. Qi J, Kim JW, Zhou Z, Lim CW, Kim B. Ferroptosis affects the progression of nonalcoholic steatohepatitis via the modulation of lipid peroxidation-mediated cell death in mice. Am J Pathol. 2020;190:68–81.

61. Yamada N, Karasawa T, Wakiya T, Sadatomo A, Ito H, Kamata R, et al. Iron overload as a risk factor for hepatic ischemia-reperfusion injury in liver transplantation: potential role of ferroptosis. Am J Transplant. 2020;20:1606–18.

68. Loguercio C, De Girolamo V, de Sio I, Tuccillo C, Ascione A, Baldi F, et al. Non-alcoholic fatty liver disease in an area of southern Italy: main clinical, histological, and pathophysiological aspects. J Hepatol. 2001;35:568–74.

73. Zhang Y, Zhao S, Fu Y, Yan L, Feng Y, Chen Y, et al. Computational repositioning of dimethyl fumarate for treating alcoholic liver disease. Cell Death Dis. 2020;11:641.

77. Zhou Z, Ye TJ, DeCaro E, Buehler B, Stahl Z, Bonavita G, et al. Intestinal SIRT1 deficiency protects mice from ethanol-induced liver injury by mitigating ferroptosis. Am J Pathol. 2020;190:82–92.

78. Zhou Z, Ye TJ, Bonavita G, Daniels M, Kainrad N, Jogasuria A, et al. Adipose-specific lipin-1 overexpression renders hepatic ferroptosis and exacerbates alcoholic steatohepatitis in mice. Hepatol Commun. 2019;3:656–69.

85. Yamada N, Karasawa T, Kimura H, Watanabe S, Komada T, Kamata R, et al. Ferroptosis driven by radical oxidation of n-6 polyunsaturated fatty acids mediates acetaminophen-induced acute liver failure. Cell Death Dis. 2020;11:144.

86. Wang Z, Hao W, Hu J, Mi X, Han Y, Ren S, et al. Maltol improves APAP-induced hepatotoxicity by inhibiting oxidative stress and inflammation response via NF-κB and PI3K/Akt signal pathways. Antioxidants. 2019;8:395.

89. Jaeschke H, Ramachandran A, Chao X, Ding W-X. Emerging and established modes of cell death during acetaminophen-induced liver injury. Arch Toxicol. 2019;93:3491–502.

93. Sun X, Niu X, Chen R, He W, Chen D, Kang R, et al. Metallothionein-1G facilitates sorafenib resistance through inhibition of ferroptosis. Hepatology. 2016;64:488–500.

99. Doll S, Proneth B, Tyurina YY, Panzilius E, Kobayashi S, Ingold I, et al. ACSL4 dictates ferroptosis sensitivity by shaping cellular lipid composition. Nat Chem Biol. 2017;13:91–98.

100. Feng J, Lu P-Z, Zhu G-Z, Hooi SC, Wu Y, Huang X-W, et al. ACSL4 is a predictive biomarker of sorafenib sensitivity in hepatocellular carcinoma. Acta Pharmacol Sin. 2020;42:160–70.
